# Itaconate as an inflammatory mediator and therapeutic target in cardiovascular medicine

**DOI:** 10.1042/BST20210269

**Published:** 2021-10-19

**Authors:** Marina Diotallevi, Faseeha Ayaz, Thomas Nicol, Mark J. Crabtree

**Affiliations:** British Heart Foundation Centre of Research Excellence, Division of Cardiovascular Medicine, Radcliffe Department of Medicine, John Radcliffe Hospital, University of Oxford, Oxford OX3 9DU, U.K.

**Keywords:** cardiovascular disease, immunometabolism, inflammation, itaconate, metabolic reprogramming, oxidative stress

## Abstract

Inflammation is a critical component of cardiovascular disease (CVD), encompassing coronary artery disease (CAD), cerebrovascular events and heart failure and is the leading cause of mortality worldwide. In recent years, metabolism has been placed centrally in the governance of the immune response. Termed immunometabolism, immune cells adapt cellular metabolic pathways to meet demands of activation and thus function. This rewiring influences not only the bioenergetics of the cell but altered metabolites act as signalling molecules to regulate cellular response. In this review, we focus on the TCA cycle derivative, itaconate, as one such metabolite with promising immunomodulatory and therapeutic potential in inflammatory cardiovascular disease.

## Itaconate

In 1836, Samuel Baup discovered an unknown product following the thermal decomposition of citric acid [1]. Four years later, Gustav Crasso synthesised the same compound by decarboxylation of *cis*-aconitate naming it itaconate — an anagram of aconitate [2]. Remarked even by Hans Krebs as a noteworthy metabolite, itaconate was nonetheless largely forgotten until the next century. In the polymer industry, itaconate has been widely used since the 1960s due to its reactive methylene group, with the 40 000 tons of it currently being produced per year [3]. However, the first suggestions of itaconate's role in immunity only came a decade ago when groups described the production of itaconic acid in LPS-activated RAW264.7 macrophages and the VM-M3 macrophage-like cell line [3–5]. Importantly, in 2013, Michelucci et al. [6] demonstrated that the IRG1 gene, which is almost exclusively expressed in immune cells, was responsible for itaconate production in LPS-activated or inflammatory macrophages. Since this discovery, research into itaconate's function has been fervently expanded.

## Itaconate as a biomarker of inflammation

Linking metabolism and immunity, itaconate is one of the most de novo synthesised metabolites in activated macrophages, reaching up to millimolar concentration intracellularly hence making it an ideal biomarker of inflammation [7]. Itaconate has been detected in the plasma of patients suffering from inflammatory disorders such as rheumatoid arthritis [8], in patients rejecting renal allografts [9], but also in urine samples from patients suffering overactive bladder syndrome [10]. Using itaconate level as an early diagnostic tool has also been suggested due to its presence in the plasma of pregnant women suffering diabetes mellitus and likely to develop complications [11].

On the contrary, itaconate was down-regulated in patients with the auto-immune disease, systemic lupus erythematosus [12]. Similarly, despite being a marker of activated immune cells following *in vitro* stimuli, itaconate was detected at very low levels in plasma and urine samples of patients suffering severe inflammation and septicaemia [13]. These differences suggest an important role for itaconate within the immune system and raises questions for understanding its molecular and physiological roles in more detail. Early on, it was demonstrated that itaconate had antibacterial properties due to its ability to inhibit the glyoxylate shunt pathway used for survival by pathogens once inside the host [14,15]. However, pathogens have evolved to use itaconate for their own needs either by forming biofilms making them even more resistant [16] or by directly metabolising it into pyruvate or acetyl-CoA [17]. Moreover, IRG1 (the gene encoding cis-aconitate decarboxylase, the protein responsible for the synthesis of itaconate), can also be induced by non-pathogenic stimuli and is detected in hepatocytes following ischemia reperfusion injury [8], in glioma [18], and by air pollution (particulate matter) in inflamed lungs [19]; these all suggest a deeper immunomodulatory role of itaconate.

## Itaconate mediates SDH inhibition

**Figure 1.**
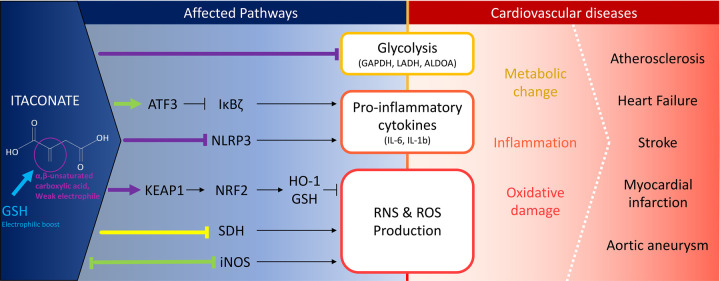
Targets of itaconate (chemical structure shown) or its derivatives (not shown) through thiol-Michael addition (—), structural competition (—) or unknown mechanism (—) and their potential role in cardiovascular diseases. The boost of electrophilicity through GSH is also illustrated.

After its high expression was revealed in inflammatory macrophages, the ability of itaconate to finely modulate the TCA cycle in response to damage and injury was discovered. Macrophages, defined by their metabolic signature, enjoy a remarkable plasticity allowing them to adapt swiftly to their environment. Similar to the Warburg effect observed in cancer cells and following inflammatory stimuli, macrophages reduce the oxidative phosphorylation (OXPHOS) route to depend primarily on the glycolytic and pentose phosphate pathways (PPP) for ATP production [20,21]. This dramatic metabolic switch including the cessation of OXPHOS stems from a dual break in the TCA cycle, hypothesised initially to be at isocitrate dehydrogenase [22]. The first break reroutes aconitate conversion to itaconate which, due to its structural similarity to succinate, inhibits succinate dehydrogenase (SDH), forming the second TCA cycle break (Figure 1).

This was demonstrated by Cordes et al. [23] in both primary macrophages and RAW264.7 cells where a strong correlation between itaconate and succinate accumulation appeared following LPS stimulation. Similarly, using LPS-stimulated bone marrow-derived macrophages (BMDMs) from Irg1^−/−^ KO mice, a significant decrease in succinate levels was detected. This was rapidly explained by the structural similarity between succinate and itaconate and because both were localised in the same compartment within the mitochondrial matrix [23]. At the same time, Lampropoulou et al. [24] also identified the post-transcriptional inhibitory role of itaconate towards SDH using a different approach. LPS-stimulated BMDMs from IRG1^−/−^ mice were treated with dimethyl itaconate, a permeable version of itaconate, demonstrating an accumulation of succinate while pro-inflammatory cytokines (IL6, IL1β, IL12) and oxidative response markers (nitrite and HIF-1α) were decreased. More importantly, left untreated, Irg1^−/−^ KO BMDMs had significantly increase levels of fumarate and malate, testifying of SDH activity to promote oxidation of succinate to fumarate in the absence of itaconate. Although a decrease in pro-inflammatory cytokines was observed, TNF-α, a target of nuclear factor kappa b (NF-κB), was not affected suggesting that NF-κB was regulated independently of itaconate, a finding later confirmed by Bambouskova et al. [25].

With the pro-inflammatory signalling roles of succinate already described, in particular its contribution to the induction of IL1β expression via HIF-1α stabilisation leading to ROS production, the role of itaconate as an immunomodulatory metabolite started to unravel [26]. In fact, inhibition of SDH has been associated with protection against ischemia-reperfusion and brain damage following heart attack and stroke, making itaconate a potential therapeutic target [27–29]. Earlier this year, Cordes et al. [30] demonstrated that itaconate inhibits SDH in a dose-response manner, and it is this exact transient inhibition of SDH activity that allows the accumulation of succinate but prevents further oxidation by SDH through reverse electron transfer from mitochondrial complex II to complex I, shielding against ROS damage.

## Itaconate modulates the KEAP1/NRF2 pathway

Not long after the discovery of the SDH-inhibitory role of itaconate, another fascinating property of itaconate was identified: its capacity for directly modifying other proteins by covalent alkylation. Itaconate contains an electrophilic α,β-unsaturated carboxylic acid that can alkylate protein cysteine residues by Michael addition. This amino acid is known to be a strong nucleophile due to its sulfur group, especially when in the thiolate form, and can be easily targeted by reactive oxygen and nitrogen species, potentially forming disulfide bonds and thus modifying protein function [31,32]. Despite being a weak electrophile generated in the matrix of the mitochondria, itaconate can use other TCA carriers such as citrate and oxoglutarate transporters to reach the cytosol where it can react with a variety of molecules [32]. To confirm this hypothesis, Mills et al. [32] developed a cell-permeable derivative of itaconate keeping its electrophilic feature and referred to as 4-octyl itaconate (4-OI) to treat HEK cells previously transfected with Kelch-like ECH-associated protein (KEAP1). KEAP1 is known to be alkylated on its sensor cysteines, preventing it from being associated with the antioxidant transcriptional factor Nrf2. Mills et al.[32] demonstrated that 4-OI could alkylate five cysteines of KEAP1 including cysteine 151 (Cys151), the main regulatory sensor for Nrf2 control. By alkylating Nrf2, 4-OI lead to an anti-inflammatory response and hallmarks of inflammation were decreased including nitrite levels, iNOS, IL10 and IL1β expression as well as ROS species. In fact, under normal conditions, KEAP1 associates to Nrf2 driving it to its degradation by ubiquitination and thus preventing it from moving into the nucleus where it then interacts with specific regulatory sequence, the antioxidant response elements (ARE), localised on the promoter of its target genes. Once produced, these targets control various antioxidant pathways such as glutathione (GSH) synthesis and regeneration and NADPH production [33,34]. Down-regulation of the Nrf2 pathway has been associated with inflammation, hypertension, diabetes; all of which are cardiovascular disease risk factors.

Further molecular analysis comparing itaconate to its derivatives have shown that endogenous itaconate does not alkylate KEAP1 like 4-OI and others as previously assumed, and more importantly, that itaconate is also less electrophilic than its derivatives [35]. However, a recent study presented evidence of endogenous itaconate activating Keap1–Nrf2 response in inflammatory conditions by pre-reacting with GSH cysteine forming GSH-itaconate (or GSH-DI) boosting its electrophilic capacity (Figure 1) [25,36].

## Itaconate modulates glycolysis

In addition to altering the KEAP1–NRF2/ARE pathway, itaconate or its derivatives can also contribute to the metabolic rewiring described earlier by directly modifying different cysteines of glycolytic enzymes including lactate dehydrogenase A (LDHA), fructose-bisphosphate aldolase A (ALDOA) and glyceraldehyde 3-phosphate dehydrogenase (GAPDH) leading to impairment of the overall glycolytic process (Figure 1) [32,37,38]. 4-OI alkylates GAPDH on its Cys22 inhibiting its enzymatic activity which consists of converting glyceraldehyde 3-phosphate to D-glycerate 1,3-bisphosphate, an irreversible metabolic switch in glycolysis [37]. Alkylation of GAPDH was also confirmed using endogenous itaconate [36]. Interestingly, Irg1^−/−^ BMDMs had a significant increase in GAPDH activity, a characteristic of activated macrophages in comparison with WT BMDMs following LPS stimulation and had increased levels of lactate and extracellular acidification rate (ECAR), two markers of an up-regulated glycolysis and inflammatory response. Unsurprisingly, the level of IL1β was also significantly increased [37]. To get a larger information of itaconate targets, Qin et al. [38] developed a novel thiol-reactive probe, 1-OH-Az, for quantitative and site-specific profiling of itaconate modified proteins and found that 260 were potentially itaconate-modified in RAW264.7 cells. Although due to technical limitations KEAP1 and GSH were not detected, the probe identified modification on Cys73 and Cys339 on ALDOA, and Cys84 on LDHA, with both undergoing inhibition of their enzymatic activity. Interestingly, Mills et al. [36] too previously identified that LDHA was alkylated on its Cys84 by 4-OI in LPS-treated macrophages, a finding later confirmed using endogenous itaconate.

## Itaconate and inflammation

Until recently, itaconate inflammatory roles were observed indirectly through glycolysis, SDH inhibition and KEAP1/NRF2 action. Lately, however, direct evidence of endogenous itaconate to modulate inflammation have emerged. Itaconate induce the protein ATF3 which in turn inhibit IκBζ, a regulator of inflammation interacting with NF-κB family (Figure 1) [25]. Since IκBζ is part of the second transcriptional response to TLR activation, it explains, in fact, why IL6, IL12 and other pro-inflammatory cytokines are decreased by itaconate but not TNF-∝, which is primarily transcribed, as discussed previously.

This year, the role of endogenous itaconate to activate signal 2 of the inflammasome, NLRP3, following LPS-tolerance in macrophages, leading to a decrease in IL1β, was also demonstrated [35,36]. This decrease is independent of SDH inhibition, NRF2 and ATF3 signalling pathways. Specifically, itaconate modifies Cys77 of gasdermin-D (GSDMD) which play a role in oligomerisation and pore formation with Caspase-1 to induce pyroptosis, an important cell death occurring in highly inflamed cells and to which IL1β secretion relies. This decrease in IL1β during late inflammasome activation was shown to be dependent on the presence of both inducible nitric oxide synthase (iNOS) and IRG1 [36].

Indeed, we have recently revealed the interplay between iNOS and IRG1 [39–41]; while studying the effects of NO in BMDMs using iNOS KO mice, we discovered that the depletion of NO caused a striking increase (10-fold) in itaconate levels following LPS/IFNγ stimulation [39], compared with cells isolated from NO-replete WT mice. Interestingly, most of the studies described herein have reported decreased levels of nitrite following itaconate treatment or IRG1 induction. These results point to a complementary co-regulation between NO and itaconate to orchestrate the response to damage and infection, which could play an important role in in the treatment of CVDs where both oxidative stress and inflammation can lead to further vascular damage. NO is also produced by endothelial NOS (eNOS) within the vessels, and plays a central role in the cardiovascular system as a signalling molecule, supporting vasodilation. Studies have shown its role in reducing hypertension and others CVDs [42]. Many clinical trials have tried in various ways to increase the uptake of NO with the aim of improving the repair and function of vessels. Nitrite therapy has been reported to improve left ventricle function in ischemia-induced heart failure through Nrf2-dependent signalling and increased antioxidant proteins [43]. On the other hand, inflamed resident macrophages in vessels can also produce large quantity of NO through iNOS during certain heart conditions. Interestingly, recent studies have measured the release of large quantities of itaconate by activated macrophages [30]. It is therefore conceivable that resident macrophages in vessels could also release large quantity of itaconate playing a role in resolution and repair of vessels by modulating NO or vice-versa.

## Itaconate in cardiovascular disease

Oxidative stress and inflammation are closely linked, since oxidative stress leads to inflammation, which itself can induce oxidative stress resulting in injury to cells [44]. In the last few years, a multitude of evidence for itaconate dampening ROS, lowering inflammation (NLRP3, IKKB) and altering metabolism has been revealed. The majority of cardiovascular diseases, including atherosclerosis, MI, stroke demonstrate a perturbation of redox homeostasis due to an in imbalance of ROS/RNS formation and ROS degrading systems which lead to an accumulation of superoxide, hydrogen peroxide, peroxynitrites [45–47]. Although canonically considered as being caused by deposition and accumulation of LDL particles in arterial intima, much evidence supports the entwined nature of inflammation with atherogenesis. LDLs are cholesterol rich particles encased in phospholipid layer with apolipoprotein B coiled around it [48]. Many pathways including the generation of ROS can lead to the modification of LDL particles and although experimental research supports the hypothesis that oxidised LDL exert pro-atherogenic effects, it remains to be conclusively proven and therapies targeting oxidative pathways have not yet been successful [49,50]. Increased ROS and oxidised lipoproteins can lead to many biological actions including damage to endothelial cells, their up-regulation of adhesion molecules (VCAM-1 and ICAM1), secretion of chemoattractants (IL8, MCP-1, etc.) as well as recruitment of circulating monocytes which differentiate into inflammatory macrophages to release pro-inflammatory cytokines (IL1β, IL18, IL8, TNF-∝) and promote atherosclerotic plaque development [51]. Furthermore, high TLR4 expression in monocytes leads to their polarisation to activated macrophages, and such expression is also associated with unstable plaque (and heart failure after MI) [52,53]. Other immune cells such as dendritic cells and T helper cells are also key in exacerbation of atherosclerosis [54].

Plaque rupture can then trigger acute thrombosis and lead to artery occlusion to cause MI. Ordinarily in the heart, ∼95% of ATP generation is sourced from OXPHOS with FAO contributing 70–90% of cardiac ATP, the remaining produced largely via glucose oxidation, lactate and less so ketone bodies and amino acids. Following myocardial injury (MI), oxidative metabolism of these substrates is suppressed and anaerobic glycolysis activated; glycolysis becomes the major supplier of ATP with stored glycogen supplying and nourishing the increased glycolysis [55]. These metabolic changes can determine the extent of cardiac damage. Here, increased glycolysis is associated with inflammation and, as detailed previously, itaconate inhibits the glycolytic pathway. The inflammatory response triggered by dead myocardial cells increases infarct size with ischemia exacerbating myocardial inflammation. Inflammatory macrophages initially phagocytose necrotic cardiomyocytes, which is followed by anti-inflammatory signalling, resulting in a metabolic shift towards a reparative macrophage phenotype. Finally, the maturation phase involves the remodelling of the extracellular matrix — here, maladaptive cardiac remodelling leads to the development of heart failure [56].

Another CVD, abdominal aortic aneurysm (AAA), is characterised by aortic wall expansion due to altered ECM integrity, macrophages are one of the first leukocytes which migrate to the aorta following injury, releasing the cytokines, collagenases and elastases needed for aneurysm formation [57]. Song et al. [58] observed inhibition of angiotensin II-induced AAA formation in ApoE^−/−^ mice after exogenous addition of 4-OI. The study demonstrated, although not exclusively, that itaconate and Nrf2 are essential to diminish the AAA formation, with 4-OI addition leading to the up-regulation of Nrf2 and subsequent decrease in pro-inflammatory cytokines (IL6, IL1β).

In addition to hyperlipidaemia, hypertension and hyperglycaemia are among prime CVD risk factors also closely linked to inflammation. Much evidence shows the inflammatory response in both hypertension and hyperglycaemia is dependent on oxidative stress [59]. Altered shear stress mechanical forces, angiotensin II, aldosterone, and cytokines in hypertension stimulate enzymes such as uncoupled nitric oxide synthase, NADPH oxidases (NOX) and the mitochondria to produce ROS [60]. NOX-derived superoxide can inactivate NO to form peroxynitrites, leading to impaired endothelial cell vasoregulation and enhanced inflammation [61].

The most compelling evidence for the potential of anti-inflammatory therapy in CVDs came from the CANTOS trial in 2017 [66]. In this trial, over 10 000 patients, with history of MI and CRP levels >2 mg/L, were treated with canakinumab, an anti-IL1β monoclonal antibody. At 3.7 years median treatment, canakinumab treatment resulted in reduction in IL6 and CRP levels by up to 40–60% as well as a reduction in major CV events by 15% overall and 25% in patients with CRP <2 mg/L — overall mortality was not reduced [66]. This was followed by the COLCOT study in 2019 which investigated the effects of colchicine in 2366 patients recruited within 30 day of having an acute MI. Colchicine (0.5 mg) daily treatment resulted in 31% lower relative risk of CVD endpoint compared with placebo with median 28.6 months follow-up and reduced risk of atherosclerotic cardiovascular disease (ASCVD) by 23% compared with placebo and in coronary artery disease (CAD) patients; CRP levels were not measured in this trial [67]. Colchicine and canakinumab are both theorised to act to inactivate the NLRP3 inflammasome which when activated could lead to caspase 1-dependent release of IL1β. As mentioned previously, there is evidence to show itaconate also modulates and interacts with NLRP3 and IL1β. Other anti-inflammatory drugs which have been or are currently being trialled for treatment of cardiovascular diseases are collated in this review [69].

## Itaconate as a potential therapeutic target

With its anti-inflammatory and oxidative stress lowering properties, itaconate is a promising candidate in the treatment of cardiovascular inflammation. Although, there are currently no active clinical trials investigating itaconate or its derivatives in any inflammatory conditions, this is most probably since research into its therapeutic use is in early stages (Table 1). However, there are several other electrophilic compounds approved for therapeutic use including: dimethyl fumarate (DMF) for plaque psoriasis and multiple sclerosis, bardoxolone for chronic kidney disease and sulforaphane for prostate cancer and asthma [62]. To note, similarly to itaconate, DMF is thought to activate the NRF2-Keap1 signalling pathways as well as an adduct to GAPDH resulting in inhibition of glycolysis and hence immune cell activation. Through this, DMF inhibits glycolysis and limits immune cell activation [63]. Although not prescribed for cardiovascular diseases, with a similar proposed mechanisms of action as itaconate, the approval of these electrophiles for therapeutic use in conditions with an inflammatory component is encouraging for itaconate.

**Table 1:** Therapeutic evidence of itaconate and its derivatives in (cardio)-vascular models

Model	Treatment	Outcome	Ref.
Heart — Ischemia-Reperfusion Injury in mice	Intravenous injection of Dimethyl itaconate (4 mg/kg/min) 10 min before or during ischemia	• Myocardial infarct size was reduced • Diminution of ROS	[24]
Heart — Acute MI model in rats	Subcutaneously Implanted of Dimethyl itaconate loaded PCL nanofibers patches Vivo on the epicardium over the infarcted region	• Myocardial protection: Tissue repair • Improvement of Left Ventricle function • Reduction in infarct area	[70]
Heart – Mouse Model of Acute Myocardial Infarction	Intramyocardial injection of N-isopropylacrylamide-co-itaconic acid (NIPAM-IA) with cardiac stromal cells	• Promotes heart repair via angiogenesis • Apoptosis inhibition • Improved cardiac stem cell retention	[71]
Vessels – Human Umbilical Vein Endothelial Cells (HUVECS)	Pre-treatement for 1 h with 4-OI (25 µM), followed by high glucose (40 mM)	• Inhibits high glucose-induced ROS production • Lipid peroxidation • Mitochondrial depolarisation	[66]
Vessels – Abdominal Aortic aneurysm model in mice	Pre-Intraperitoneal injection of 4-OI (50 mg/kg) before, during and after Angiotensine II induction	• Inhibition of angiotensin II-induced abdominal aortic aneurysm (AAA) formation in ApoE^−/−^ mice; via activation of Nrf2	[58]
Vessels – Transient middle cerebral artery occlusion mice model	Dimethyl itaconate (20 mg/0.5 ml saline per mouse) via intraperitoneal route at the beginning of occlusion	• Decreases neurologic deficit score and toxic conversion of microglia • Improve neurologic function • Decrease in pro-inflammatory cytokine: IL1β	[64]
Vessels – Mice model of cerebral ischemia reperfusion	Itaconic acid was infused for 30 min at 15 mg/kg/min prior to ligation, and for 30 min during reperfusion	• Protects against GSH depletion and improves the antioxidant capacity of cells • Improved arterial blood flow • Preserved cerebral oxygen tension	[65]
Cardiotoxicity – Acute Myocardial Infarction caused by cancer drug (Doxorubin)	Dimethyl itaconate administration at first 4 days with 100 mg/kg per day since DOX intraperitoneal injection	• Inhibition of oxidative stress by altering Nrf2/HO-1 • Diminish acute cardiotoxicity	[67]
Metabolism – Hyperlipidaemia in rats	Itaconate solution to drink instead of water	• decreased visceral fat in rats • Decrease in free fatty acid and triglycerides • Inhibition of glycolysis	[68]

A role of itaconate as a preventive and protective drug, with its ability to enhance repair and lessen oxidative stress, is starting to emerge, whether it is by modulating Nrf2 or through SDH-inhibition (Table 1). One of the pioneering studies indicating that itaconate could have therapeutic interest came from mice treated intravenously with DI 10 min before and during ischemia, which had reduced myocardial infarct size compared with mice treated with saline solution. The same study showed that hypoxic neonatal rat cardiomyocytes following an *in vitro* simulation of MI injury were more protected from hypoxia-induced cell death if pre-treated with 0.25 and 1 mM DI [24]. Soon after, the protective effects of DI were replicated in cerebral IR injury [64]. Treatment with DI at the beginning of transient middle cerebral artery occlusion in mice also resulted in a significant decrease in neurologic deficit score, inhibition of microglia toxic conversion as well as reduced IL1β protein expression. Last year, Cordes et al. [65] demonstrate that itaconate-mediated SDH inhibition lead to a reduction in reactive species and an increase in glutathione helping brain tissue recovery by pre-treating with itaconate for 30 min prior to ligation, and again for 30 min, an hour after ligation a mouse model of cerebral reperfusion injury with ischemia or traumatic brain injury. Similarly, pre-treatment with 4-OI potently inhibited high glucose-induced cytotoxicity in human umbilical vein endothelial cells, a cell model use to study the vasculature system [66]. Finally, using itaconate as a co-treatment to dampen cardiotoxicity in cancer therapy was also evidenced [67]. Mice given dimethyl itaconate had better recovery following treatment with doxorubicin, an antitumor drug with strong effects such as arrhythmia leading to potential heart failure, hence providing evidence for the detoxifying role of itaconate. There are several studies investigating itaconate therapy in the heart, although more research is clearly needed; for example, despite the availability of the IRG1^−/−^ mouse, no studies have interrogated the potential therapeutic effects of itaconate in models of atherosclerosis.

Through itaconate's role in the rewiring of the glycolytic pathway, shifting the metabolic profile of inflammatory macrophages towards the reparative macrophage phenotype is another avenue to explore in the therapeutic potential of the itaconate pathway. In one study linking itaconate and hyperlipidaemia, dietary-controlled mice fed with itaconate solution instead of water had decreased levels of visceral fat through glycolysis suppression, as well as a reduction in FFA and triacylglycerols in plasma by 33% and 50% respectively [68]. In the context of smoking as another leading cause of CVD mortality, 4-OI intervention of acute lung injury reduced lung inflammation and oxidative stress the cellular level as well as up-regulating expression of NRF2 and RF2 target genes in lung tissue and RAW264.7 cells [69].

Excitingly, some groups have also started using itaconate directly as a biomaterial to produce scaffolds and other clinical devices to aid the cardiovascular regeneration and repair following injury. For instance, PCL nanofibers loaded with dimethyl itaconate, subcutaneously implanted into the infarct area initially maintained inflammation through suppression of anti-inflammatory activity whilst later promoting anti-inflammatory activity and tissue repair. This resulted in overall myocardial protection with reduced infarct area and improved ventricular function [70]. The patches also increased expression of MCPIP-1, a negative regulator of oxidative stress. Similarly, taking advantage of itaconate's proficiency as a polymer, injectable hydrogels using hydrophilic and negatively charged itaconate properties were created forming poly(N-isopropylacrylamide-co-itaconic acid) (NIPAM) to treat MI [71]. These NIPAM-gels, once delivered, were favourable for maintaining high viability of cardiac stem cells (CSCs), promoting their proliferation and facilitating the formation of spheroids, which in turn released growth factors promoting neonatal rat cardiomyocytes activation and survival. After injection of these itaconate-microgels into mice, the heart started the repairing process through angiogenesis and inhibition of apoptosis. These studies as indicate the versatility of itaconate as well the importance of the drug delivery method in developing itaconate as a therapeutic (rather than preventative) treatment.

## Perspectives

- Targeting inflammation has promise to be an effective strategy in the treatment of cardiovascular diseases, the leading collective cause of mortality worldwide.
- Immune cell metabolite itaconate modulates several pathways involved in oxidative stress, inflammation and glycolysis all of which are hallmarks of cardiovascular disease.
- Elucidating further the mechanistic role of itaconate and its true inflammatory nature are needed as well as translational and clinical studies in cardiovascular diseases to ascertain its true therapeutic potential.

## Open Access

Open access for this article was enabled by the participation of University of Oxford in an all-inclusive *Read & Publish* pilot with Portland Press and the Biochemical Society under a transformative agreement with JISC.

## Abbreviations

4-OI: 4-octyl itaconate
AAA: abdominal aortic aneurysm
BMDMs: bone marrow-derived macrophages
CAD: coronary artery disease
CVD: cardiovascular disease
DMF: dimethyl fumarate
GAPDH: glyceraldehyde 3-phosphate dehydrogenase
GSH: glutathione
KEAP1: Kelch-like ECH-associated protein
LDHA: lactate dehydrogenase A
NIPAM: N-isopropylacrylamide-co-itaconic acid
NOX: NADPH oxidases
OXPHOS: oxidative phosphorylation
SDH: succinate dehydrogenase
